# Natural antibodies drive type 2 immunity in response to damage-associated molecular patterns

**DOI:** 10.1172/jci.insight.177230

**Published:** 2024-03-12

**Authors:** Arlind B. Mara, Kavita Rawat, William T. King, Claudia V. Jakubzick

**Affiliations:** Department of Microbiology and Immunology, Geisel School of Medicine at Dartmouth, Hanover, New Hampshire, USA.

**Keywords:** Immunology, Adaptive immunity, Allergy

## Abstract

Allergic airway disease (AAD) is an example of type 2 inflammation that leads to chronic airway eosinophilia controlled by CD4 Th2 cells. Inflammation is reinforced by mast cells and basophils armed with allergen-specific IgE made by allergen-specific B2 B cells of the adaptive immune system. Little is known about how AAD is affected by innate B1 cells, which produce natural antibodies (NAbs) that facilitate apoptotic cell clearance and detect damage- and pathogen-associated molecular patterns (DAMPS and PAMPS). We used transgenic mice lacking either B cells or NAbs in distinct mouse models of AAD that require either DAMPS or PAMPS as the initial trigger for type 2 immunity. In a DAMP-induced allergic model, driven by alum and uric acid, mouse strains lacking B cells (CD19^DTA^), NAbs (*Ig*^HEL^ MD4), or all secreted antibodies (*sIgm*^–/–^*Aid*^–/–^) displayed a significant reduction in both eosinophilia and Th2 priming compared with WT or *Aid*^–/–^ mice lacking only germinal center–dependent high-affinity class-switched antibodies. Replenishing B cell–deficient mice with either unimmunized B1 B cells or NAbs during sensitization restored eosinophilia, suggesting that NAbs are required for licensing antigen-presenting cells to prime type 2 immunity. Conversely, PAMP-dependent type 2 priming to house dust mite or *Aspergillus* was not dependent on NAbs. This study reveals an underappreciated role of B1 B cell–generated NAbs in selectively driving DAMP-induced type 2 immunity.

## Abstract 2

Natural antibodies play a critical role in driving DAMP-induced type-2 inflammation in allergic airway disease mouse models.

## Introduction

Asthma affects approximately 300 million people worldwide and is responsible for an estimated 250,000 deaths each year (1). Allergic asthma is characterized by airway obstruction, caused by smooth muscle constriction, mucus production, and chronic airway inflammation, predominantly driven by Th2 cells of the adaptive immune system. It is further enhanced by allergen-specific IgE, which arms innate effector type 2 immune cells and DCs for increased allergen capture (2–7). A critical aspect of the pathology of allergic asthma is the abundance of airway eosinophils, which contribute to many key alterations, including the formation of mucus plugs and epithelial damage in the airways (8–11).

B cells have a well-established role in allergic airway diseases (AADs), primarily through producing allergen-specific class-switched immunoglobulins IgE and IgG1 in an IL-4–dependent manner. These high-affinity antibodies are generated mainly from conventional “B2” B lymphocytes as part of a germinal center reaction and prompt mast cell degranulation and basophil activation, reinforcing the inflammatory response (12–18). However, the involvement of B2 B cells and their secreted antibodies in the emergence of late-stage airway eosinophilia remains controversial, with conflicting reports on their necessity (19–28). While it is widely accepted that activation of Th2 cells is critical for eosinophilic inflammation, it was previously thought that B cells do not play a significant role in initiating Th2 cell priming but are necessary for subsequent Th2 cell expansion in conditions of limited allergen exposure (29, 30).

One reason for the controversy on B cells in AAD stems from the wide use of the muMT mouse model, which lacks mature B cells owing to a disrupted immunoglobulin mu chain gene (31). Recent findings by our group suggest that this model has concealed the important role of B cells in priming type 1 immune responses in a variety of models (32, 33). The controversy on B cell involvement in experimental AAD also stems from model discrepancies. For instance, the widely used alum-OVA mouse model involves sensitizing mice through i.p. injection of the antigen OVA emulsified in aluminum hydroxide (alum adjuvant), followed by airway OVA challenge. This model primarily relies on the adjuvanticity of uric acid, an endogenous damage-associated molecular pattern (DAMP), triggering DC maturation and licensing these cells for priming adaptive Th2 responses (34). Not only is uric acid released after alum administration, uric acid crystals injected i.p. are able to mimic all Th2 adjuvant activities of alum, and mice developed AAD (35). Other AAD models, such as those induced by the natural allergens house dust mite (HDM) or *Aspergillus fumigatus*, much more depend on the adjuvanticity of pathogen-associated molecular patterns (PAMPs) recognized by pattern recognition receptors on host cells, although they concomitantly also release DAMPs like uric acid (35–38). These insights highlight the need to reevaluate the function of B cells in various type 2 immunity models and call for the use of alternative B cell–deficient mouse models to obtain a more accurate understanding.

Natural antibodies (NAbs) are germline-encoded, low-affinity immunoglobulins produced without explicit antigenic stimulation (39–44). Predominantly of the IgM and IgG types, NAbs also include IgA and IgE (45, 46). They recognize conserved microbial determinants, offering an early broad defense against pathogens and facilitating downstream adaptive immunity (47–54). Notably, NAbs have been shown to bind to DAMPs, with the prevailing view being that they help clear out excessive DAMPs associated with cell death, thereby dampening inflammation and preventing the onset of autoimmunity (55–59). NAbs are primarily synthesized by B1 B cells, a specific subset of B lymphocytes enriched in the peritoneal and pleural cavities and spleen, carrying a repertoire of specificity that develops in early life without prior immunization. Whereas our group has recently shown that NAbs derived from B1 cells play an essential role in priming type 1 immune responses to a variety of model neoantigens and tumors (32, 33, 60), very little attention has been given to a potential role of NAbs in the induction of type 2 immunity. Guided by our recent work, where we described better mouse models to study B cell biology in disease, we focused on determining whether NAbs play a critical role in triggering type 2 immunity using DAMP- and PAMP-induced AAD as the readout model. Our experiments revealed that, while B1 cells and NAbs are crucial in alum and uric acid–driven AAD, they are not as relevant in the context of HDM and *Aspergillus*-driven disease, explaining at least in part why B cell involvement in AAD has been so controversial.

## Results

### DAMP-induced airway eosinophilia and antigen-specific CD4^+^ T cell priming is impaired in B cell–deficient mice.

To probe for the involvement of B cells and various types of antibodies in type 2 immunity and AAD, we employed several mouse stains lacking NAbs, conventional high-affinity antibodies, or B cells altogether. Unlike muMT mice, which are devoid of B cells, transgenic Ig^HEL^ mice possess circulating B cells, with over 90% specificity for hen egg lysozyme (HEL) due to expression and allelic exclusion of a transgenic BCR. This high specificity leads to a markedly reduced diversity of NAbs and other antigen-specific antibodies, providing a unique model to study the effects of limited antibody repertoire in disease contexts (61). Moreover, in Ig^HEL^ mice, the presence of B cells maintains the integrity of the lymph node architecture, including the B cell zone and its surrounding lymphatics, supporting optimal DC and T cell migration, which are critical for Th2 immunity to develop but are severely compromised in the commonly used muMT mice (62). Additionally, Ig^HEL^ mice do not exhibit the immunological compensatory mechanism observed in muMT mice, which leads to excessive development of plasmacytoid DCs that bias interpretation (32). It has indeed been shown that plasmacytoid DCs heavily influence AAD development both at the priming and challenge phase of the response (63, 64). Considering these factors, Ig^HEL^ mice were chosen as a better alternative mouse model for exploration of the role of B cell–derived antibodies in eosinophilic inflammation in 4 distinct models of AAD: alum-OVA, uric acid–OVA, HDM, and *Aspergillus fumigatus*. We chose these models because they are established and widely used to induce AAD and type 2 immunity. Specifically, the alum-OVA formulation, which is free of PAMPs, induces immune responses through the release of the DAMP uric acid. In contrast, while the HDM and *A*. *fumigatus* models also partly depend on DAMPs to initiate type 2 inflammation, the presence of PAMPs in these models and their significant role in triggering subsequent type 2 immune responses renders them suitable surrogate models for PAMP-induced AAD (35, 36, 65). Using these mechanistically distinct AAD models allows us to evaluate the specific contributions of B1 B cells and NAbs in the onset of DAMP-induced AAD.

**Figure 1:**
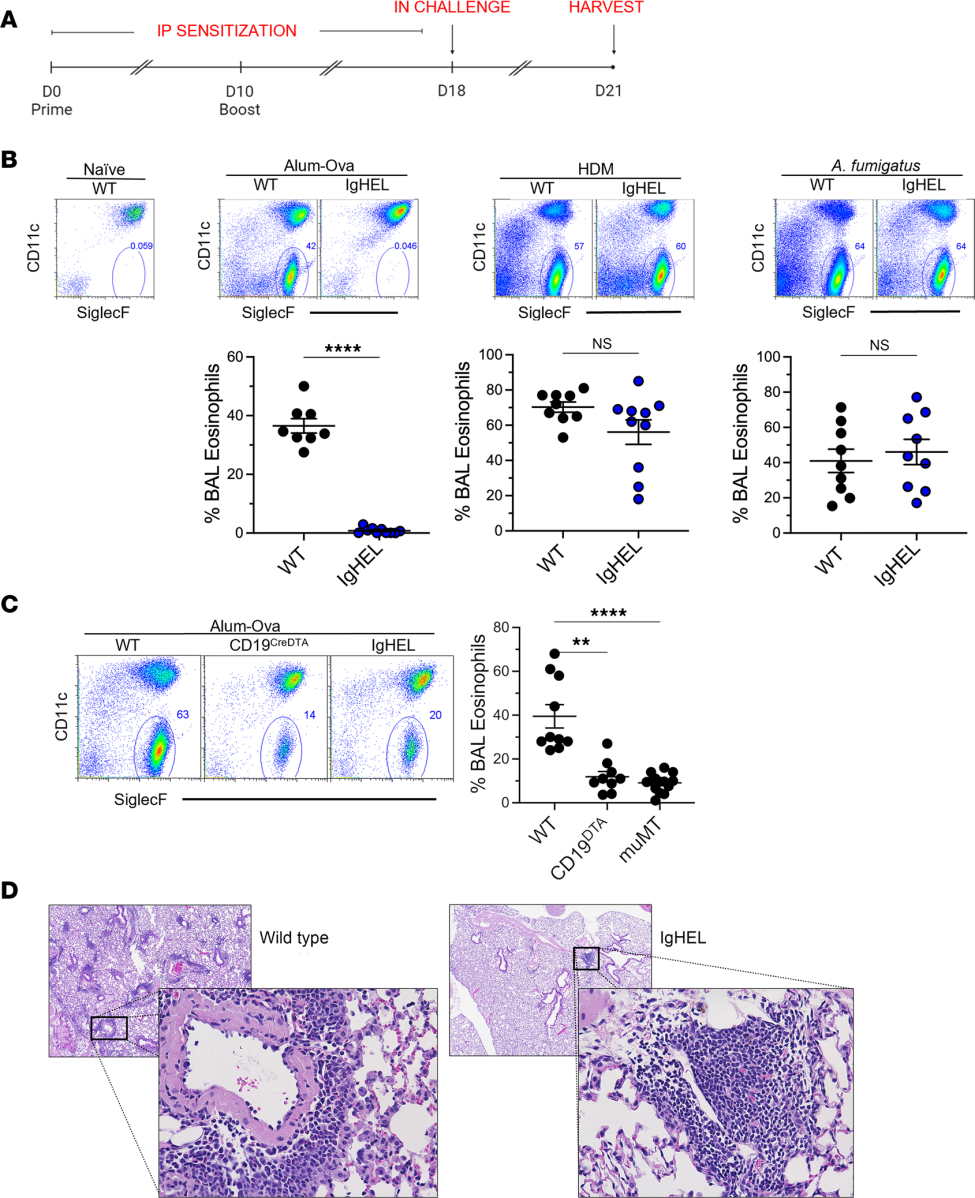
B cell–deficient mice develop PAMP-induced, but not DAMP-induced, allergic airway disease. (**A**) Illustration of experimental timeline. (**B**) Representative flow cytometry plots and scatter plot graphs of eosinophils (defined as SSC^hi^, CD11b^+^, CD11c^–^, SiglecF^+^, Ly6G^–^) in bronchoalveolar lavage fluid (BALF) collected from WT or Ig^HEL^ mice following induction of AAD with alum + OVA, house dust mite (HDM), or *A*. *fumigatus*. (**C**) Representative flow cytometry plots and scatter plot graph of eosinophils in BALF collected from WT, CD19^DTA^, or muMT mice following induction of AAD. (**D**) Representative H&E micrographs presenting lung histopathology following alum-OVA AAD induction in WT and Ig^HEL^ mice. Original magnification, ×100 (low-power image); ×400 (high-power image). Data are shown as the mean ± SEM. Each point represents data from an individual animal, with data are pooled from 2 independent experiments per graph. Statistical comparisons were performed in GraphPad Prism using a Mann Whitney *U* test when comparing 2 groups and a Kruskal-Wallis ANOVA on ranks followed by a Dunn’s post hoc test for multiple comparisons with WT control when 3 or more groups were compared. **P < 0.01, ****P < 0.0001.

One hallmark feature of AAD is the increased presence of eosinophils in the airways. Because activated CD4 Th2 cells are essential for the development of this type of eosinophilic inflammation, we concentrated on this aspect in our study across all 3 models. In each model, mice were sensitized by i.p. injections on days 0 and 10, followed by intranasal challenge on day 18. Three days after challenge (day 21), bronchoalveolar lavage (BAL) fluid and lung tissue samples were collected to assess airway eosinophilia (see timeline in Figure 1A). By flow, alveolar macrophages are CD11c^+^SiglecF^+^ and eosinophils are CD11c^–^SiglecF^+^ (Figure 1B). Surprisingly, in the DAMP-induced, alum-OVA AAD model, Ig^HEL^ mice did not develop eosinophilic inflammation when compared with WT mice (Figure 1B). However, both WT and Ig^HEL^ mice exhibited airway eosinophilia in the PAMP-containing HDM- and *A*. *fumigatus*–AAD models (Figure 1B). Similarly, observations made in other B cell–deficient muMT and CD19^DTA^ mice indicated that they do not develop significant airway eosinophilia using the alum-OVA model (Figure 1C). In the alum-OVA AAD model, histological examination of the lungs in WT mice revealed widespread perivascular and peribronchiolar inflammation characterized by the presence of macrophages, lymphocytes, and eosinophils, which are typical features of AAD (Figure 1D). In contrast, Ig^HEL^ mice displayed infrequent lymphocytic aggregates that were not always localized around vessels or bronchioles and lacked eosinophils but demonstrated some degree of organization reminiscent of tertiary lymphoid tissues (Figure 1D and Supplemental Figure 1; supplemental material available online with this article; https://doi.org/10.1172/jci.insight.177230DS1).

**Figure 2:**
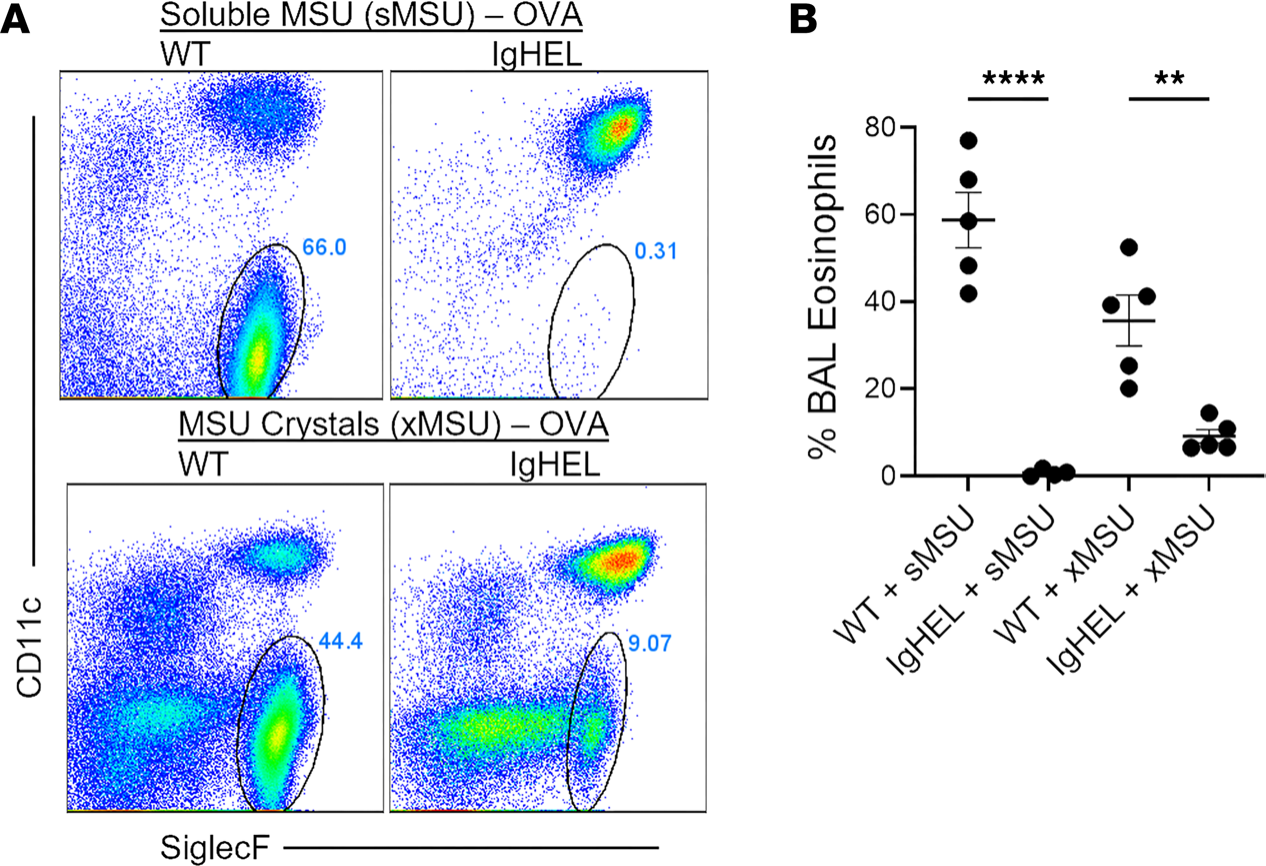
Ig^HEL^ mice do not respond to the adjuvanticity of uric acid, an important endogenous DAMP. (**A**) Representative flow cytometry plots and (**B**) scatter plot graphs of eosinophils (defined as SSC^hi^, CD11b^+^, CD11c^–^, SiglecF^+^, Ly6G^–^) in BALF collected from WT or Ig^HEL^ mice following induction of AAD using soluble monosodium urate (sMSU) or monosodium urate crystals (xMSU) as the Th2-potentiating adjuvant. Data are shown as the mean ± SEM. Each point represents data from an individual animal. Statistical comparisons were performed in GraphPad Prism using Kruskal-Wallis ANOVA on ranks followed by a Dunn’s post hoc test for multiple comparisons. **P < 0.01, ****P < 0.0001.

The enhancement of Th2 immune responses by alum has been linked to the release of uric acid, a well-known endogenous DAMP associated with various inflammatory conditions (66). In humans, only crystallized uric acid has adjuvant properties, whereas in rodents, both crystallized and soluble forms can act as proinflammatory DAMPs, with potency equal to alum for inducing type 2 immunity (35, 67, 68). To address another DAMP-driven type 2 model, Ig^HEL^ mice were i.p. sensitized with either uric acid crystals and OVA or soluble uric acid and OVA. Then, mice were challenged intranasally with OVA (same timeline as Figure 1A). Consistent with results of the alum-OVA model, Ig^HEL^ mice sensitized with either the soluble or crystallized forms of uric acid plus OVA, did not exhibit airway eosinophilia to the extent seen in WT controls (Figure 2). These findings collectively underscore the importance of B cells in the development of airway eosinophilia within AAD models that depend on the adjuvanticity of DAMPs.

**Figure 3:**
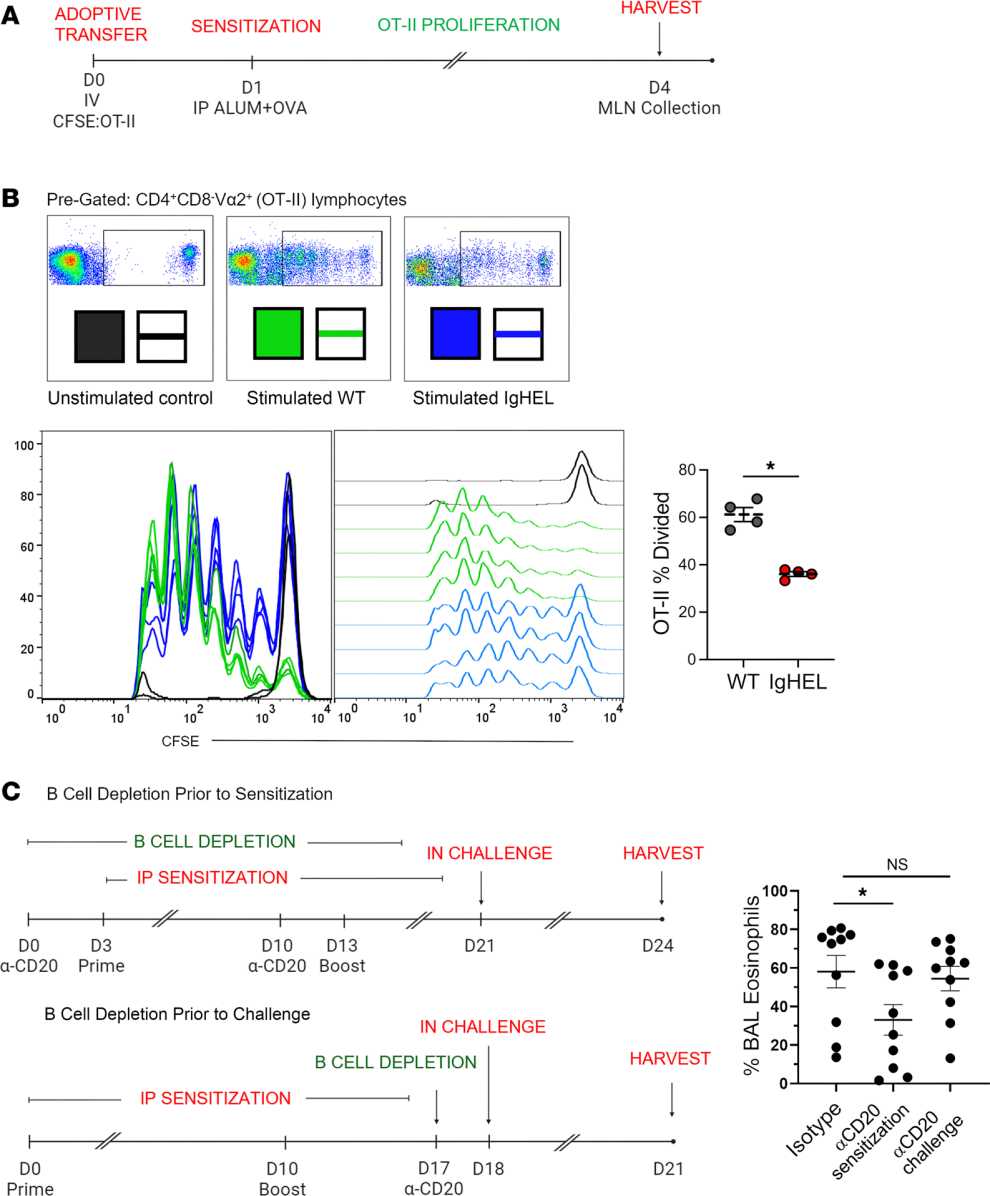
Antigen-specific CD4^+^ T cell priming is impaired in B cell–deficient mice. (**A**) Illustration of experimental timeline. (**B**) Representative flow cytometry histograms indicating differences in OT-II cell proliferation in WT and Ig^HEL^ mice following administration of alum-OVA. (**C**) Illustration of experimental timeline of depletion of B cells via α-CD20 performed prior to sensitization or prior to challenge. Scatter plot graph of eosinophils in BALF of WT mice that were treated with B cell–depleting anti-CD20 antibody (clone MB20-11), or isotype control, during sensitization or challenge phases of AAD induction with the alum-OVA model. Data are shown as the mean ± SEM. Each point represents data from an individual animal. Statistical comparisons were performed in GraphPad Prism using a Kruskal-Wallis ANOVA on ranks followed by a Dunn’s post hoc test for multiple comparisons with isotype control when 3 or more groups were compared. Mann-Whitney *U* was used for comparing OT-II cell proliferation between 2 groups. *P < 0.05.

Given that antigen-specific Th2 cells are necessary for the development of airway eosinophilia in AAD models, we investigated whether the absence of functional B cells and NAbs in the alum-OVA model would lead to reduced antigen-specific T cell proliferation. This would highlight a role for B cells in the initial priming of helper T cells. CFSE-labeled OVA-specific CD4^+^ T (OT-II) cells were adoptively transferred into both WT and Ig^HEL^ mice, followed by an i.p. injection of alum-OVA 1 day after transfer (Figure 3A). Four days after immunization, T cell proliferation was assessed in the mediastinal lymph nodes. WT mice showed significantly more proliferation of OT-II T cells compared with Ig^HEL^ mice (Figure 3B and Supplemental Figure 2A), suggesting that a diverse repertoire of B cells is necessary for the initial priming of antigen-specific T cells during the sensitization phase of DAMP-induced AAD. Similar findings to the Ig^HEL^ mice were observed in CD19^DTA^ mice, which lack B cells compared with WT and *Aid^–/–^* mice, which have hyper-IgM syndrome and lack high-affinity, isotype-switched antibodies (Supplemental Figure 2B).

To further test the hypothesis that B cells are necessary for Th2 cell priming rather than merely for their reexpansion upon challenge, B cells were depleted using an anti-CD20 antibody (clone MB20-11) during either the sensitization phase or the challenge phase (Figure 3C). B cell depletion at the time of sensitization alone led to a reduction in the severity of airway eosinophilia, whereas their depletion at the time of challenge had no effect (Figure 3C). However, the effects of B cell depletion via anti-CD20 were not as profound as those observed in IgHEL Ig^HEL^, muMT, or CD19^DTA^ mice. This is most likely due to the fact that CD20 is downregulated during plasma cell differentiation; therefore, anti-CD20 antibody treatment only mildly depletes plasma cells that develop from B1 cells. Moreover, anti-CD20–treated mice are expected to have residual NAb concentrations that could account for the observed differences.

### Functional B cell reconstitution in B cell–deficient mice rescues airway eosinophilia in DAMP-induced AAD.

In our DAMP-induced model, absence of airway eosinophilia was observed in B cell–deficient or NAb-deficient mice. To determine if this condition was reversible, we conducted adoptive transfers of WT B cells into Ig^HEL^ mice, which led to a partial restoration of eosinophilia. Nevertheless, the resulting eosinophilic inflammation did not achieve the levels observed in WT control mice (Supplemental Figure 3A), likely due to the limited B cell reconstitution in Ig^HEL^ mice, in which the B cell niche is predominantly occupied by HEL-specific B cells. Indeed, only a slight increase in the frequency of WT B cells was detected in Ig^HEL^ mice after transfer of WT B cells (Supplemental Figure 3B). The observed partial rebound in eosinophilia in Ig^HEL^ mice with few WT B cells following alum-OVA administration attests to the fact that even small numbers of B cells make a substantial contribution to the onset of DAMP-induced AAD.

**Figure 4:**
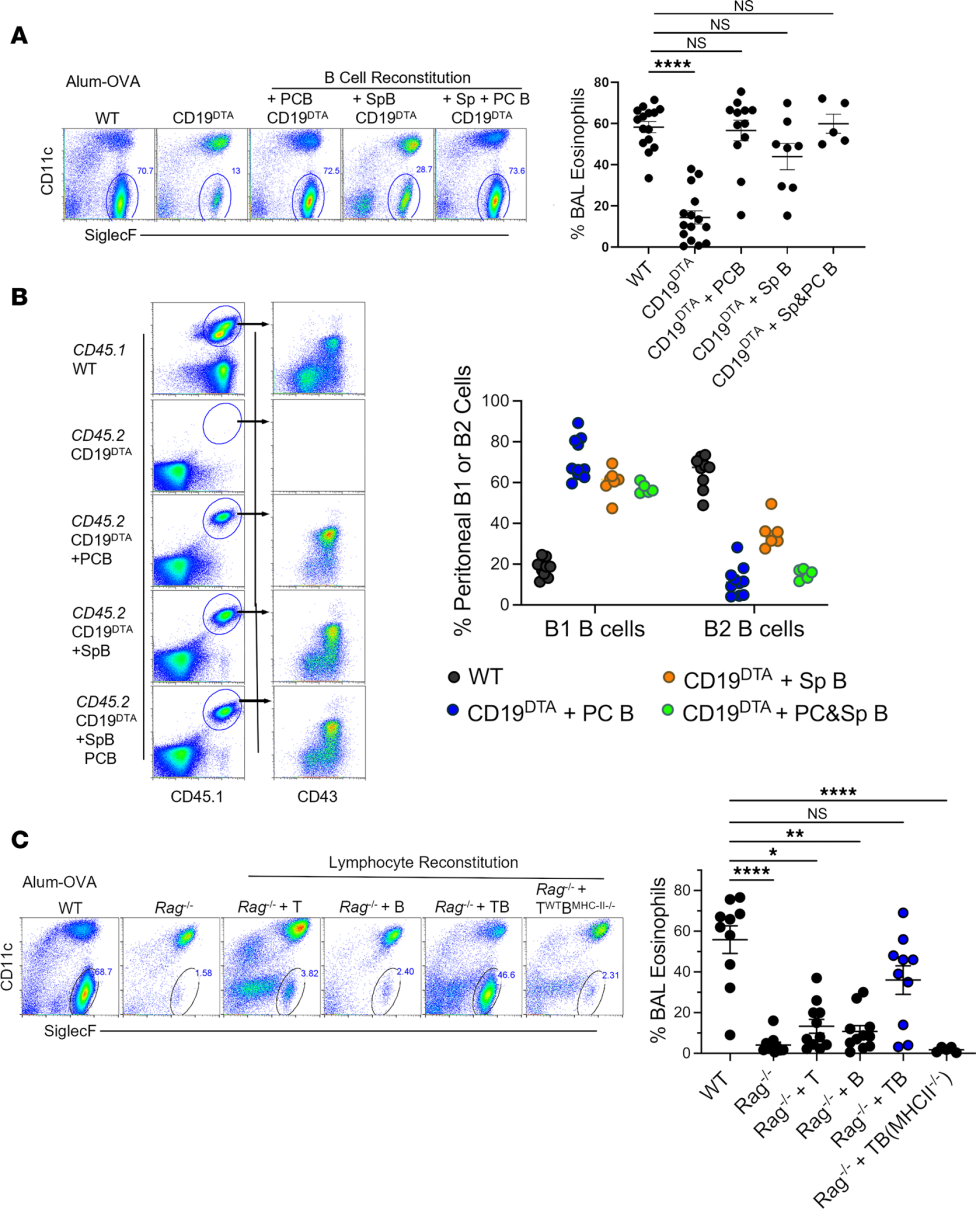
Reconstitution with functional B cells rescues development of alum-OVA AAD in B cell–deficient mice. (**A**) Representative flow cytometry plots and scatter plot graphs of numbers of eosinophils in BALF of WT, CD19^DTA^, and CD19^DTA^ mice reconstituted with B cells following alum-OVA AAD induction. (**B**) Flow plots and scatter plot graph showing frequency of B1 B and B2 B cell reconstitution in the peritoneal cavity of reconstituted CD19^DTA^ mice. (**C**) Representative flow cytometry plots and scatter plot graphs of numbers of eosinophils in BALF of WT, Rag^–/–^, and B, T, or B and T cell–reconstituted Rag^–/–^ mice flowing alum-OVA AAD induction. Data are shown as the mean ± SEM. Each point represents data from an individual animal. Data are pooled from 2 independent experiments. Statistical comparisons were performed in GraphPad Prism using and a Kruskal-Wallis ANOVA on ranks followed by a Dunn’s post hoc test for multiple comparisons with WT control. *P < 0.05, **P < 0.01, ****P < 0.0001.

To address the challenges of B cell reconstitution in Ig^HEL^ mice, we conducted experiments in CD19^DTA^ and *Rag*^–/–^ mice, in which the B cell niche is open and more readily reconstituted. Like Ig^HEL^ mice, CD19^DTA^ mice exhibited significantly reduced airway eosinophilia compared with WT controls (Figure 1B and Figure 4A). The adoptive transfer of WT B cells successfully reconstituted a considerable proportion of the B cell niche in CD19^DTA^ mice (Figure 4B and Supplemental Figure 4A). Moreover, this reconstitution led to the restoration of airway eosinophilia in CD19^DTA^ mice to levels similar to WT mice, regardless of whether the B cells originated from the peritoneal cavity, spleen, or both (Figure 4A). These findings emphasize the necessary role of B cells in DAMP-induced AAD, particularly highlighting that reconstitution with B1 B cells was predominantly achieved in the peritoneal cavity and to a lesser extent B2 B cells (Figure 4B), suggesting that B1 B cells alone might be sufficient to rescue the AAD phenotype in B cell–deficient mice.

Further experiments in *Rag*^–/–^ mice, which lack mature B and T cells, reinforced the essential role of B cells. When *Rag*^–/–^ mice were reconstituted with WT-derived B cells, T cells, or a combination thereof (Figure 4C), and cell fractions were prepared to avoid contamination (Supplemental Figure 4B), only those reconstituted with both WT T and B cells developed airway eosinophilia comparable to that of WT mice (Figure 4C). In contrast, *Rag*^–/–^mice reconstituted with WT T cells and then with B cells from MHCII^–/–^ mice hardly showed eosinophilia (Figure 4C). These results support the notion that B cells are instrumental in the development of DAMP-induced AAD, but we could not conclude if B cells were required as MHCII antigen-presenting cells or provided the crucial source of NAbs.

### MHCII expression on B cells is required for the development of eosinophilic inflammation in DAMP-induced AAD.

**Figure 5:**
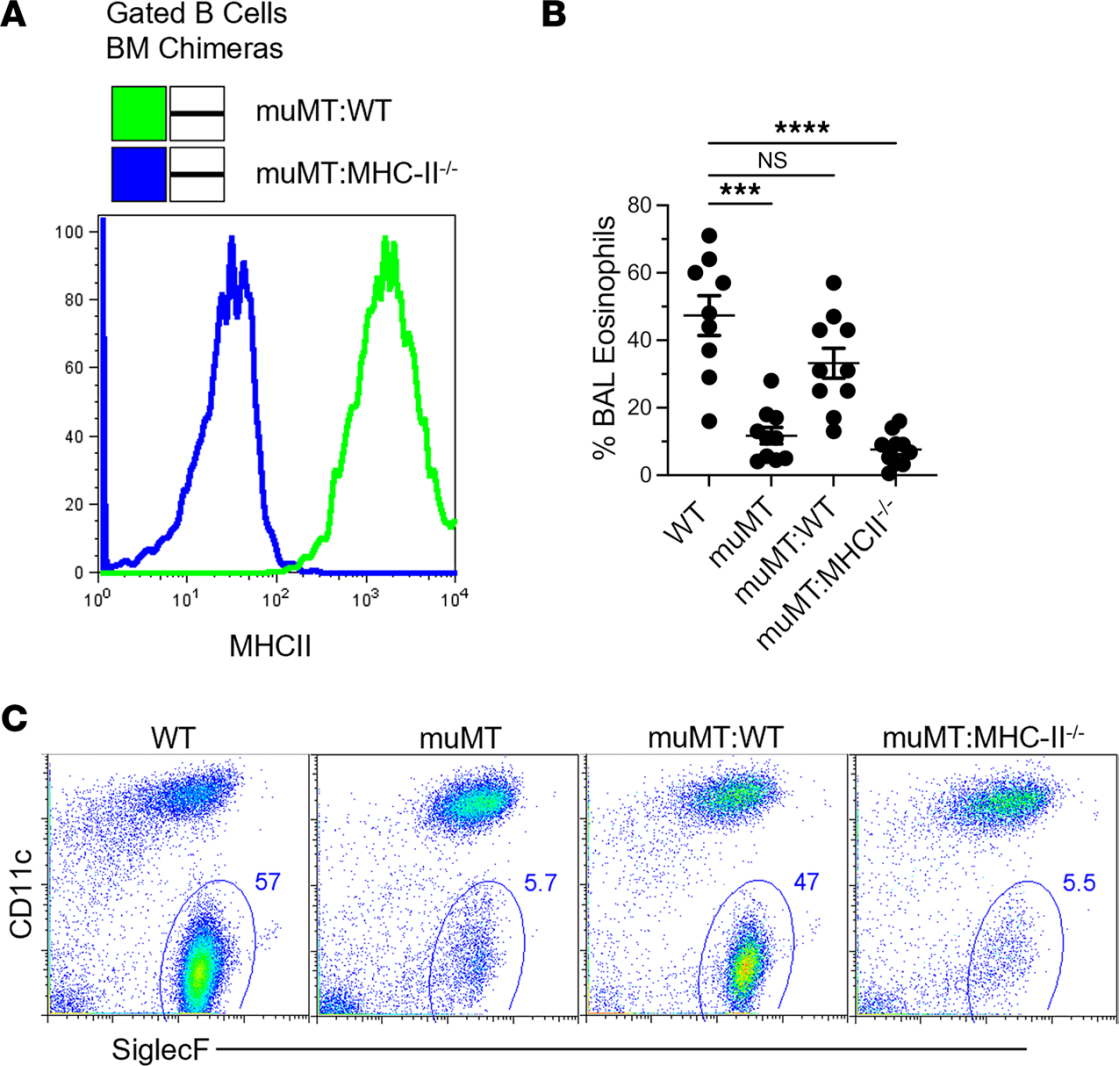
MHCII-expressing B cells are required for the development of DAMP-induced AAD. (**A**) Histogram overlay illustrating B cell phenotypes of generated bone marrow chimera mice. (**B**) Representative flow cytometry plots and (**C**) scatter plot graph of eosinophils in BALF of animals following induction of AAD using the alum-OVA model. Data are shown as the mean ± SEM. Each point represents data from an individual animal. Data are pooled from 2 independent experiments. Statistical comparisons were performed in GraphPad Prism using and a Kruskal-Wallis ANOVA on ranks followed by a Dunn’s post hoc test for multiple comparisons with WT control. ***P < 0.001, ****P < 0.0001.

To evaluate the requirement for MHCII on B cells in driving airway eosinophilia within the alum-OVA model, we engineered transgenic bone marrow chimeras. These chimeras were designed so that B cells were MHCII deficient, while the majority of DCs retained MHCII expression, using a 90:10 mix of bone marrow from muMT:MHCII^–/–^ mice transplanted into irradiated WT recipients (Figure 5A). The absence of MHCII expression on B cells, or the complete lack of B cells, resulted in a marked decrease in airway eosinophilia (Figure 5, B and C). This most likely reflects a loss of Th2 priming. More studies are required if this is related to a defect in IL-4^+^ T follicular helper (Tfh) cell development, since early Tfh cell development that depends on antigen presentation by cognate B cells presenting antigen to naive T cells can be conducive to Th2 development (28, 30). Alternatively, MHCII expression on B1 B cells might also be essential for their transition into NAb-producing plasma cells, as has been recently demonstrated, further highlighting a role for NAbs (66).

### Circulating NAbs are essential for inducing airway eosinophilia in DAMP-induced AAD.

It is known that MHCII deficiency in B cells also interferes with the ability of B1 cells to produce NAbs and affects the capability of B2 cells to undergo class-switch recombination, which is necessary for generating allergen-specific IgG1/IgE antibodies (69). These factors could significantly influence the development of AAD, yet their potential roles have not been not fully explored in previous studies. To effectively determine the specific role of B cells and their secreted immunoglobulins in triggering eosinophilic inflammation in AAD, we again used our alum-OVA model in 3 transgenic mouse strains with varying B cell–related deficiencies: Ig^HEL^ mice (lacking a broad repertoire of antibodies and NAbs but retaining quasi-monoclonal B cell numbers), *Aid^–/–^* mice (which have hyper-IgM syndrome and a NAb repertoire, with functional BCRs and antigen presentation ability, but lack germinal center–dependent isotype switching and affinity-matured antibodies, such as IgG1 and IgE, implicated in AAD), and *sIgm^–/–^Aid^–/–^* mice (agammaglobulinemic mice lacking all circulating NAbs and antigen-specific antibodies but with retained functional BCRs and antigen presentation capacity of B cells).

**Figure 6:**
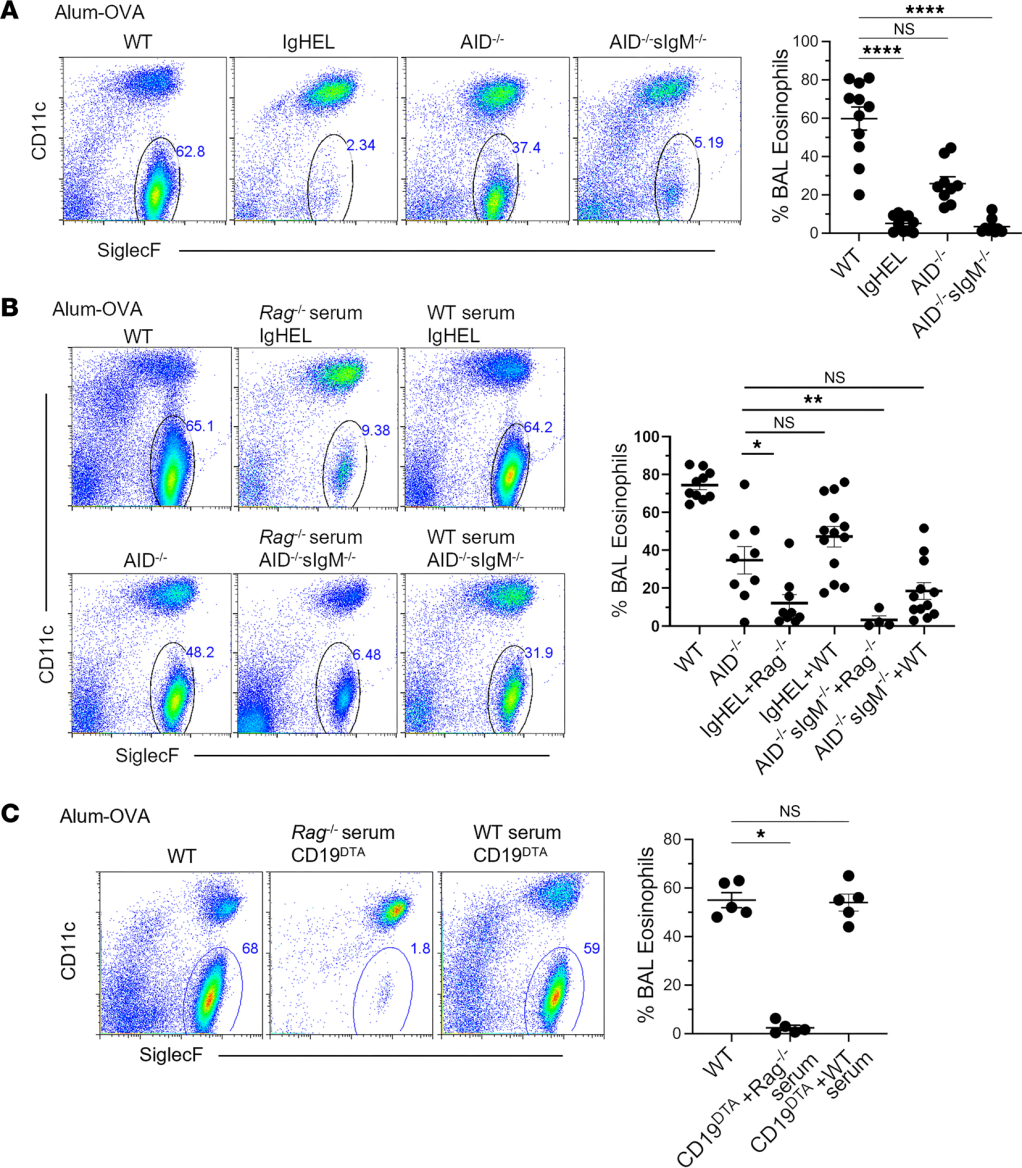
Natural antibodies play a critical role in the induction of DAMP-driven AAD. (**A**) Representative flow cytometry plots and scatter plot graphs of numbers of eosinophils in BALF of WT, AID^–/–^, sIgM^–/–^AID^–/–^, and Ig^HEL^ mice following alum-OVA AAD induction. (**B**) Representative flow cytometry plots and scatter plot graphs of numbers of eosinophils in BALF of WT, AID^–/–^, sIgM^–/–^AID^–/–^, and Ig^HEL^, mice following i.p. alum-OVA with sIgM^–/–^AID^–/–^, and Ig^HEL^ mice also receiving admixed i.p. naive WT (to reconstitute natural antibodies) or Rag^–/–^ (control) serum at the time of sensitization. (**C**) Representative flow cytometry plots and scatter plot graphs of numbers of eosinophils in BALF of WT and CD19^DTA^ mice following i.p. alum-OVA with CD19^DTA^ mice also receiving admixed i.p. naive WT (to reconstitute natural antibodies) or Rag^–/–^ (control) serum at the time of sensitization. Each point represents data from an individual animal. Data are pooled from 2 independent experiments. Statistical comparisons were performed in GraphPad Prism using and a Kruskal-Wallis ANOVA on ranks followed by a Dunn’s post hoc test for multiple comparisons with WT control. **P < 0.01, ****P < 0.0001.

First, *Aid^–/–^* mice, lacking affinity-matured, isotype-switched allergen-specific antibodies (IgE or IgG1), developed only partially reduced airway eosinophilia compared with WT controls (Figure 6A). These findings suggest that high-affinity allergen-specific antibodies are not necessary for the development of airway eosinophilia following allergen challenge, aligning with previous reports regarding IgE antibodies (70). Agammaglobulinemic *sIgm^–/–^Aid^–/–^* mice did not develop airway eosinophilia, mirroring the observations in Ig^HEL^ mice lacking functional B cells (Figure 6A). As *sIgm^–/–^Aid^–/–^* mice still have B cells that are capable of antigen presentation, these results suggest that antigen presentation by B cells alone is insufficient to induce AAD and imply that free circulating NAbs are required to induce airway eosinophilia in the DAMP-induced AAD model.

To investigate this hypothesis, we performed adoptive transfer of unimmunized/naive WT serum into Ig^HEL^, *sIgm^–/–^Aid^–/–^*, and CD19^DTA^ mice at the time of sensitization with alum-OVA (Figure 6, B and C). Administering naive WT serum was sufficient to restore eosinophilic airway inflammation in Ig^HEL^ and *sIgm^–/–^Aid^–/–^* mice, while transferring control *Rag^–/–^* serum, which lacks circulating antibodies, did not restore eosinophilia in AAD (Figure 6B). More importantly, adoptive transfer of naive WT serum, but not naive *Rag^–/–^* serum, rescued airway eosinophilia in CD19^DTA^ mice, which lack B cells entirely, to WT levels (Figure 6C), indicating that reconstitution with NAbs alone is sufficient to rescue DAMP-induced AAD in B cell–deficient mice. Similarly, preliminary data demonstrate that naive serum from germ-free WT mice also restored AAD in CD19^DTA^ mice (Supplemental Figure 5), supporting the fundamental role of germline encoded NAbs in the development of airway eosinophilia in the DAMP-induced AAD model.

## Discussion

B lymphocytes are important immune cells that perform a range of functions, bridging both the innate and adaptive facets of the immune response. They can present antigens to CD4^+^ T cells, promoting the differentiation of Tfh cells, and produce high-affinity, antigen-specific antibodies required for both protective and allergic immune responses when they differentiate into plasma cells. B1 B cells generate germline-encoded, broadly reactive antibodies previously shown to be vital in apoptotic cell clearance, antibacterial defenses, and antitumor immunity (32, 33, 60). However, despite their versatility, the precise role NAbs play in the onset of type 2 immunity and subsequent eosinophilic inflammation during AAD remained unclear, and the overall role of B cells is still debated in the scientific literature.

It is important to note several key differences between Ig^HEL^ and CD19^DTA^ mouse models. First, complete B cell depletion in CD19^DTA^ necessitates homozygous expression of diphtheria toxin A (DTA). Second, for the use of Ig^HEL^ mice, breeding strategies must employ Ig^HEL^ female mice to prevent the passive transfer of NAbs from WT mothers to their offspring. Third, Ig^HEL^ mice may, under certain inflammatory conditions, induce the production of an endogenous B cell repertoire. Therefore, careful consideration of the chosen model is required. Measuring the ratio of transgenic Ig^HEL^ B cells (IgMa^+^ allotype) to endogenous B cells (IgMa^–^) can reveal the extent of endogenous B cell induction in specific inflammatory settings. In contrast, CD19^DTA/DTA^ mice do not exhibit the promotion of endogenous B cells during inflammation, maintaining the B cell deficiency observed in steady-state conditions and, thereby, providing a potentially more suitable model for studying the effects of B cell function in the context of robust inflammation.

In this study, we used multiple mouse models exhibiting varying degrees of absolute or functional B cell deficiency, alongside 4 mechanistically distinct models of AAD, to investigate the role of B cells in the development of type 2 inflammation. Each model investigated leads to AAD development in mice but differs in their type 2 priming and allergen sensitization mechanism. Specifically, the alum-OVA model depends on the adjuvant properties of an endogenous DAMP, uric acid. Uric acid is released after alum-triggered cell death and activates downstream Th2 cells responsible for eosinophilic inflammation in AAD, and uric acid can completely mimic the effects of alum in priming mice to develop AAD in response to OVA inhalation (34, 35). On the other hand, the HDM and *A*. *fumigatus* allergens also include PAMPs capable of directly engaging pattern recognition receptors on DCs, thus facilitating their activation (71) and priming of Th2 cells during the sensitization process. Here, we show that B cells play a critical role in the development of AAD that relies on the adjuvanticity of DAMPs for sensitization. However, B cells are relatively dispensable for the development of AAD in models that rely on the adjuvanticity of PAMPs. Mice lacking a repertoire of broadly reactive circulating NAbs (Ig^HEL^ mice) did not develop AAD in the alum-OVA model but exhibited airway eosinophilia similar to WT mice in the HDM or *A*. *fumigatus* AAD models.

NAbs are recognized for their role in binding to DAMPs, with the notion that this interaction primarily aids in DAMP clearance to mitigate excessive inflammation. However, our findings challenge this notion, suggesting that these DAMP-detecting NAbs might also play a role in triggering downstream inflammatory responses upon recognizing DAMPs. In support of this, we conducted experiments in which B cell–deficient mice were reconstituted with naive WT serum during sensitization, which effectively rescued the development of AAD in Ig^HEL^, *sIgm^–/–^Aid^–/–^*, and CD19^DTA^ mice. The evolutionary driving force for mounting type 2 immunity to DAMP recognition might be initiation of type 2 immunity, since type 2 immunity and the associated induction of ILC2s, eosinophils, and alternatively activated macrophages aids in restoration of tissue repair and homeostasis (72).

Soluble uric acid, while known for its potent antioxidant properties, can function as a robust DAMP when it crystallizes in humans (73), while both the crystallized and soluble form can activate immune responses in rodents (67). Indeed, we show that both soluble and crystallized uric acid can induce AAD in WT mice when used as an adjuvant for OVA sensitization. Studies have shown that monoclonal IgM antibodies targeting uric acid can accelerate its crystallization, thereby enhancing the adjuvant properties of this endogenous DAMP (74). Considering that the Th2-enhancing effects of the alum adjuvant are associated with uric acid, it’s plausible to hypothesize that NAbs, which recognize uric acid (34), play a critical role in activating its adjuvant capabilities by promoting its crystallization. These uric acid crystals may subsequently stimulate the maturation and activation of specialized antigen-presenting cells, ultimately priming Th2 cells in alum-OVA or DAMP-driven AAD models. Our data also indicate that the crystallization of uric acid is not the only immunopotentiating effect that NAbs are playing in this phenomenon. While crystallized uric acid slightly enhanced the ability of NAb-deficient Ig^HEL^ mice to develop airway eosinophilia in response to OVA challenge, it did not account for its rescue to WT levels, as we have observed with the serum transfer studies. It is therefore attractive to speculate that immune complex formation with uric acid crystals, allergen, and NAbs might be required to achieve downstream activation of antigen-presenting cells that prime Th2 cells, though further research will be required to test this hypothesis. Other crystals, like Charcot-Leyden crystals derived from eosinophils and Ym1 crystals derived from alternatively activated macrophages, are other forms of crystallizable DAMPs frequently observed in sites of type 2 immunity, and they have all been shown to boost type 2 immunity, while at the same time binding to IgM antibodies (75). In the context of our data, it appears that NAbs can either directly activate and license antigen-presenting cells to initiate Th2 responses, which are pivotal for orchestrating subsequent eosinophilic inflammation, but more research is needed to elucidate the precise mechanism on how NAbs can activate DCs.

Collectively, our findings indicate that NAb production by B1 B cells is required for the development of type 2 immunity driven by DAMPs. Interestingly, this process seems to be circumvented in AAD models that use allergens containing PAMPs, like HDM and *A*. *fumigatus*. In this scenario, it appears that antigen-presenting cells may be activated directly through the recognition of PAMPs. This direct activation pathway may enable the priming of Th2 cells without relying on innate, NAbs to detect DAMPs and initiate the adaptive immune response. Our findings help explain why the precise role of B cells in experimental asthma has been so elusive, since the contribution of B cells depends on the mouse model used to deplete B cells as well as the type of adjuvant that is at the heart of the type 2 immune response.

## Methods

### Sex as a biological variable

Both male and female mice were utilized in this study, as we had previously determined that no significant differences in exist between the 2 sexes regarding the outcomes reported in our manuscript.

### Mice

C57BL/6NCr (CD45.2; strain code 556) and B6-Ly5.1/Cr (CD45.1 congenic; strain code 564) mice were purchased from Charles River/NCI Grantee Program. OT-II–transgenic mice [B6.Cg-Tg(TcraTcrb)425Cbn/J, 004194], muMT (002288), *Aicda* (AID^–/–^, 008825), *Ig^HEL^*MD4 (Ig^HEL^, 002595), *Rag1*^–/–^ (008449), B6.129P2(C)-*Cd19^tm1(cre)Cgn^*/J (CD19^cre^, 006785), B6.129P2-*Gt(ROSA)26Sor^tm1(DTA)Lky^*/J (Rosa-DTA, 009669), sIgM^–/–^AID^–/–^ (generated by in-house breeding), and B6.129S2-H2dlAb1-Ea/J (MHCII^–/–^, 003584) mice were purchased from The Jackson Laboratory. *Aicda* AID^–/–^ mice B cells are unable to undergo Ig class-switch recombination and have only serum IgM in circulation, lacking all other Ig isotypes. In Ig^HEL^ mice (with a hypo-IgM repertoire), greater than 90% of IgM-secreting B cells are specific for HEL. CD19^DTA^ mice are CD19^cre^ mice crossed with Rosa^LSL–DTA^ mice twice to achieve CD19^cre+(DTA/DTA)^ mice. The Cre recombinase driven by the expression of CD19 in turn activates the expression of the DTA in the Rosa locus, which causes the specific depletion of B cells. muMT mice lack mature B cells; Rag1^–/–^ mice lack mature B and T cells; sIgM^–/–^AID^–/–^ mice lack circulating Ig (agammaglobulinemic) but retain a functional B cell receptor and ability to present antigen through MHCII. All mice were bred in-house. Mice were genotyped or phenotyped prior to studies, used at 6–8 weeks of age, and housed in a specific pathogen–free environment at Dartmouth Hitchcock Medical Center, an AAALAC-accredited institution. Mice were housed at 20°C–22°C, 30%–70% relative humidity, with ad libitum access to food and water on a standard 12-hour-light/12-hour-dark cycle.

### Bone marrow chimeras

Eight-week-old B6-Ly5.1 (CD45.1) WT mice were lethally irradiated with 2 doses of 4.6 gray 12 hours apart. Following the second irradiation, recipient mice received 5 × 10^6^ donor bone marrow cells in sterile PBS via intravenous injection into the lateral tail vein. To generate WT BM chimera mice, irradiated CD45.1 WT mice received 100% CD45.2 WT bone marrow or a 90%:10% mixture of muMT/WT CD45.2 bone marrow. To generate B cell–deficient chimera (muMT-like) mice, irradiated CD45.1 WT mice received 100% bone marrow from muMT donors. To generate BM chimera mice lacking MHCII expression in B cells, irradiated CD45.1 WT mice received a 90%:10% mixture of bone marrow from muMT/MHCII^–/–^ donors, resulting in 100% of B cells in these mice lacking MHCII expression. Sulfamethoxazole/trimethoprim antibiotic suspension was added to the drinking water of irradiated mice 1 day prior to irradiation and for 28 days after bone marrow reconstitution.

### Allergic airway disease models

#### DAMP-induced model.

Mice were sensitized via i.p. injection of 25 μg endotoxin-free OVA (MilliporeSigma A5503, >98% purity) admixed with sterile, reagent-grade aqueous aluminum hydroxide Al(OH)_3_, solubilized monosodium urate salt (MilliporeSigma) or monosodium urate crystals (InvivoGen) suspended in PBS. Mice were primed on day 0 and boosted on day 10. On day 18 after prime dosage, mice were challenged intranasally with 25 μg OVA in 50 μL sterile PBS under anesthesia with aerosolized isoflurane.

#### PAMP-induced models.

Mice were sensitized via i.p. injection of 25 μg (protein content) *D*. *pteronyssinus* (HDM) extract (Stallergenes Greer) or with 10 μg (protein content) of *A*. *fumigatus* extract (Stallergenes Greer). Mice were primed on day 0 and boosted on day 10. On day 18 after prime dosage, mice were challenged intranasally with 25 μg HDM or 10 μg *A*. *fumigatus* in 50 μL sterile PBS under anesthesia with aerosolized isoflurane.

### In vivo OT-II cell proliferation

Spleens were harvested from OT-II–transgenic mice and homogenized in sterile PBS by pushing the cells through a 70 μm nylon mesh cell strainer to obtain a single-cell suspension. The cells were stained with carboxyfluorescein succinimidyl ester (CFSE) and adoptively transferred to WT or Ig^HEL^ mice via intravenous injection. One day following adoptive transfer of the CFSE-labeled cells, mice received a single i.p. dose of alum-OVA to sensitize the animals. Four days following this injection, mice were humanely euthanized, and the mediastinal lung draining lymph node was collected and homogenized to obtain single-cell suspensions. OT-II cell proliferation was assessed via flow cytometry.

### B cell depletion

B cells were depleted using an anti-mouse CD20 monoclonal antibody (clone MB20-11, catalog BE0356, Bio X Cell), which has been shown to achieve rapid and sustained depletion of murine B cells. For depletion at the stage of sensitization, mice were i.p. injected with 5 μg anti-CD20 antibody/mouse 3 days prior to alum-OVA injection. This dose was chosen to deplete B cells during the sensitization stage, and the time between boost dose and challenge was adjusted to allow for replenishment of B cells at the time of challenge. For depletion during the challenge stage, each mouse received 250 μg anti-CD20 antibody 1 day prior to challenge. This dose was chosen to achieve rapid and sustained depletion of B cells during the challenge phase of the model.

### B and T cell reconstitution and serum rescue experiments

B and T cells were collected from spleens and peritoneal cavities from naive WT mice or MHCII^–/–^ mice by negative enrichment using the mouse Pan B or mouse Pan T cell isolation kits from Miltenyi Biotech. For adoptive cell transfer and reconstitutions, cells were transferred at a (1:1) donor-to-recipient ratio, with one-half of the dose delivered via intravenous injection and the other half delivered via i.p. injection to reconstitute both systemic and peritoneal compartments. Mice were allowed reconstitute for 4 weeks following adoptive transfers prior to undergoing induction of AAD using the alum-OVA model based on the timeline displayed in Figure 1A. For experiments involving adoptive transfer of serum, pooled sera from 5 WT or Rag1^–/–^ mice were admixed with alum-OVA and mice sensitized i.p.

### Flow cytometry

Airway (to assess eosinophilia) and peritoneal cavity cells (used for phenotyping purposes) were obtained via bronchoalveolar and peritoneal lavage. For cells obtained from tissues, tissues were minced with scissors or teased with 26-gauge needles and then digested with 2.5 mg/mL Collagenase D (Roche) for 30 minutes at 37°C. 100 μL of 100 mM EDTA was added to stop 1 mL enzymatic digestion, and digested tissue was homogenized through repetitive aspiration through a glass Pasteur pipette and then passed through a 70 μm nylon filter to acquire single-cell suspension from lung draining lymph nodes and spleen. Cells were stained with appropriate antibody cocktails containing mixtures of the following antibodies: phycoerythrin-conjugated (PE-conjugated) anti-SiglecF (clone S17007L, catalog 552126), PerCP-Cy5.5– or BV510-conjugated anti-B220 (clone RA3-6B2, catalog 103235), PE-Cy7–conjugated anti-CD11c (clone N418, catalog 117318), BUV395-conjugated anti-CD11b clone M1/70, catalog 563553), PE-conjugated anti-CD5 (clone 53-7.3, catalog 100607), allophycocyanin- or allophycocyanin-Cy7–conjugated to anti-CD19 (clone 1D3/CD19, catalog 152410), Pacific Blue or BV421 conjugated to anti-CD4 (clone GK1.5, catalog 11-0041-82), anti-CD8a (clone 53-6.7, catalog100725), anti-CD3 (clone 17A2, catalog 100333), anti-Ly6G (clone 1A8, catalog 127611), FITC conjugated to anti-Ly6G (clone18, catalog 127606), CD3 (clone 17A2, catalog 555274), CD23 (clone B3B4, catalog 101605), PE-Cy7 conjugated to anti-Vα2 TCR (clone B20.1, catalog 127882), anti-CD43 (clone S11, catalog 143209), PerCP-Cy5.5– or BV510-conjugated anti-MHCII (I-A/I-E) (clone M5/114.15.2, catalog BDB562363), BUV805 conjugated to anti-CD4 (clone GK1.5, catalog BD612898), or anti-CD8a (clone 53-6.7, catalog 110741). The viability dye DAPI (D9542, Sigma-Aldrich) was added immediately before each sample was acquired on a 5-laser BD Symphony A3 analyzer (BD Biosciences). Data were analyzed using FlowJo (Tree Star). BUV395 (Cd11b) and BUV805 (CD8a) antibodies were purchased from BD, all others were purchased from Biolegend. To determine the Th2 cell–dependent eosinophilic airway inflammation in BAL fluid, lungs were lavaged with 4 mL saline, BAL cells were counted, and eosinophils were analyzed and counted.

### Microscopy

Harvested lungs were gently perfused with PBS, inflated with 10% neutral buffered formalin, and fixed for 24 hours in 10% neutral buffered formalin. Lung tissue was dehydrated in ethanol gradients, cleared in xylene, and then paraffin embedded. Paraffin-embedded sections (5 μm in thickness) were mounted on slides and stained with H&E. Images were collected with a Keyence BZ-X800 Series digital microscope.

### Statistics

Statistical analyses were performed using GraphPad Prism. All results are expressed as the mean ± SEM; dots represent individual measurements.

An unpaired Mann-Whitney *U* test was used when comparing 2 groups of data, a 1-way ANOVA with a Tukey’s post hoc test for multiple comparisons was used when comparing 3 or more groups of data. The α value was set at 0.05, and statistical significance was denoted if the *P* value was less than 0.05.

### Study approval

All experiments and procedures involving animals were approved by the Institutional Animal Care and Use Committee of Dartmouth College.

### Data availability

The data that support the findings of this study are made available throughout the manuscript and supplemental materials. Values for all data points in graphs are reported in the Supporting Data Values file.

## Author contributions

ABM, KR, and CVJ conceptualized the project and designed experiments. CVJ and ABM obtained funding. ABM, KR, and WTK performed experiments. ABM, KR, and CVJ analyzed data. ABM and CVJ wrote the manuscript. All authors edited the manuscript.

## Supplementary Material

Supplemental data

Supporting data values

## Version 1 03/12/2024

In-Press Preview

## Version 2 04/22/2024

Electronic publication
