# Supplementary material for: Natural antibodies drive type 2 immunity in response to damage-associated molecular patterns
Source: JCI Insight. 2024 Mar 12;9(8):e177230. doi: 10.1172/jci.insight.177230 (PMC11141869; doi:10.1172/jci.insight.177230)
Supplement: Supplemental data [file jciinsight-9-177230-s148.pdf]

Supplemental Information for *Natural antibodies drive type 2 immunity in response to damage associated molecular patterns.*

Arlind B. Mara[1*], Kavita Rawat[1*], William T. King[1], and Claudia V. Jakubzick[1‡]

[1]Department of Microbiology and Immunology, Geisel School of Medicine at Dartmouth, Hanover, NH, 03756, USA

[*]Authors contributed equally to this work
[‡]Corresponding author

Correspondence:
Claudia Jakubzick, Ph.D.
Department of Microbiology and Immunology
Geisel School of Medicine at Dartmouth College
626W Borwell
One Medical Center Drive
Lebanon, NH, 03756
claudia.jakubzick@dartmouth.edu

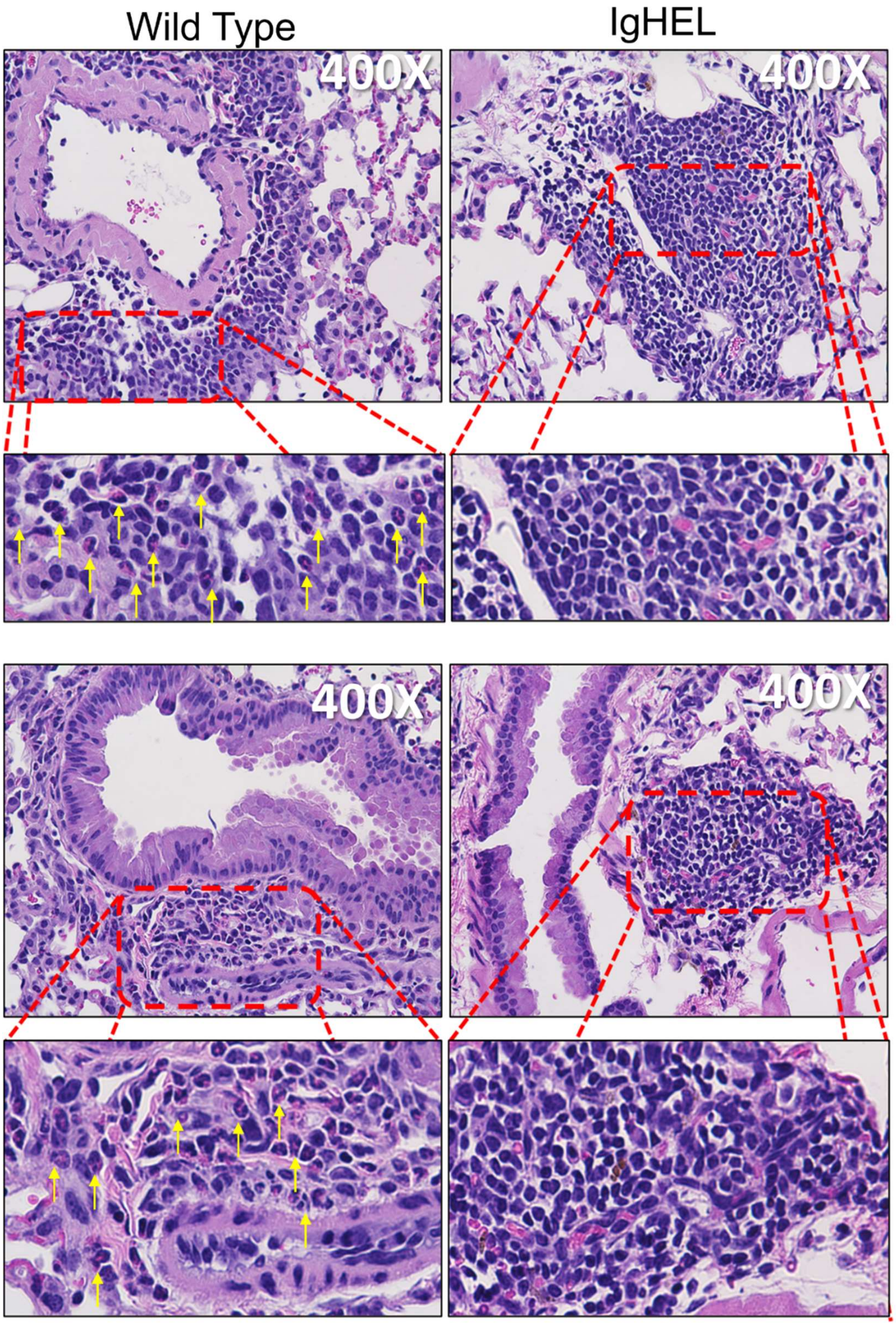

Figure S1. Close-up micrographs of H&E lung sections taken from WT and IgHEL mice following induction of AAD using the Alum-OVA model. Note the lack of peribronchiolar and perivascular eosinophilic inflammation in IgHEL mice as compared to WT controls. Yellow arrows point to areas where eosinophils can be observed.

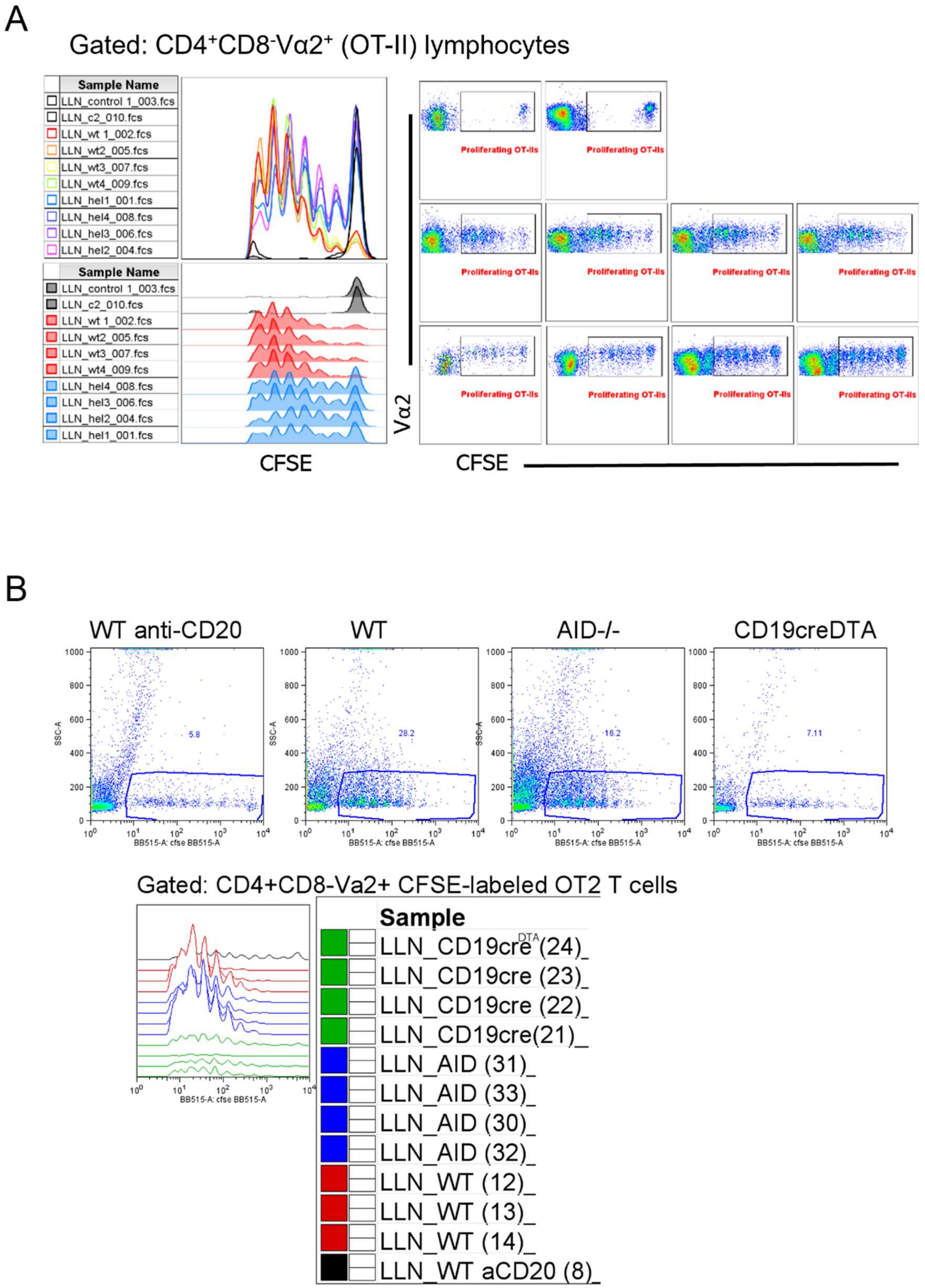

Figure S2. Raw flow cytometry data demonstrating the extent of adoptively transferred CFSE labeled OT-II cell proliferation in (A) WT and IgHEL, and (B), WT, $CD19^{DTA}$, $AID^{-/-}$, and B cell depleted WT (WT aCD20) mice stimulated with an injection of Alum-Ova.

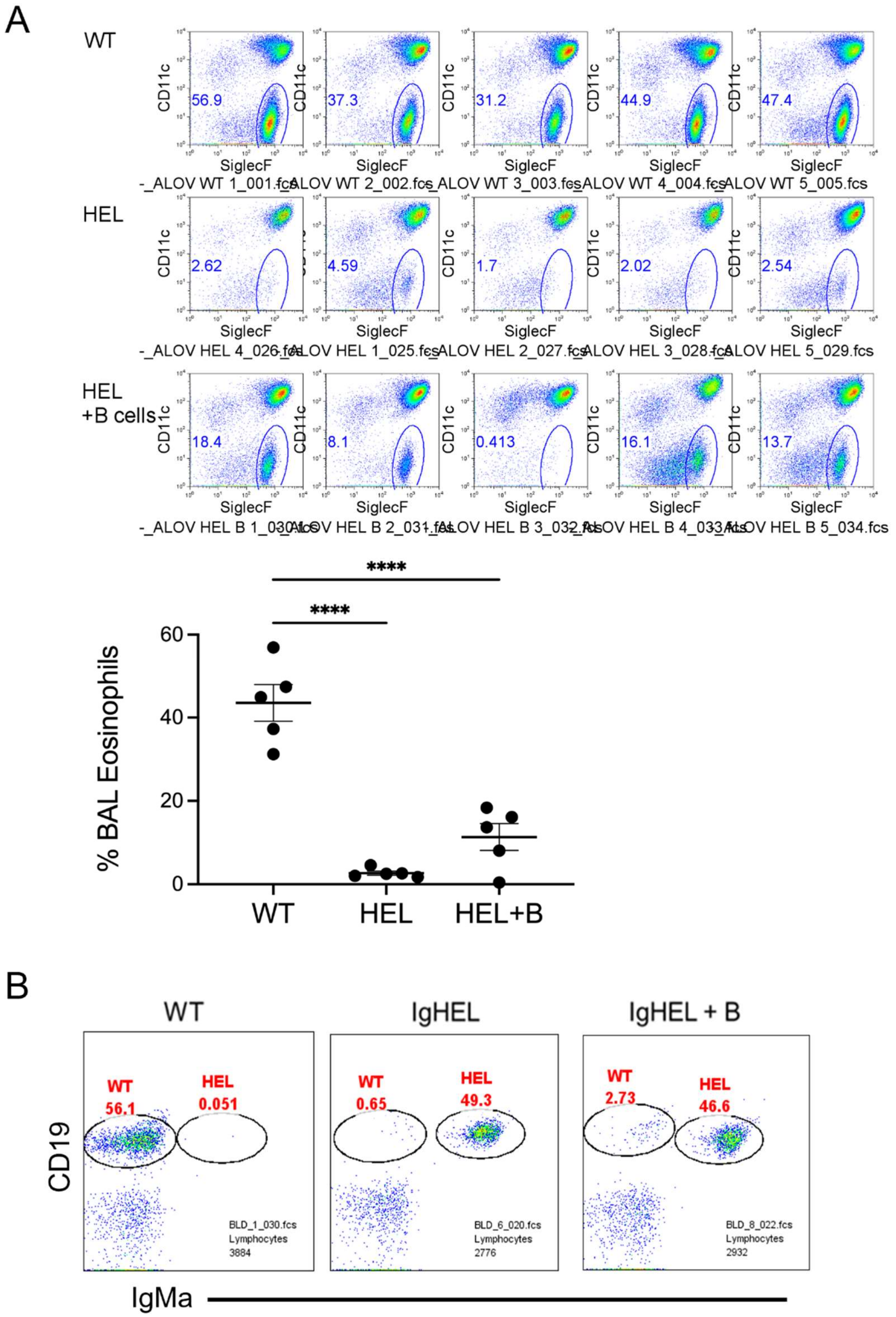

Figure S3. ***Adoptive transfer of WT B cells to IgHEL mice only marginally restores WT B cell niche and only partially rescues Alum-OVA AAD.*** (A) Representative flow cytometry plots and scatter plot graphs of eosinophils (defined as SSC^{hi},$CD11b^{+}$,$CD11c^{-}$,$SiglecF^{+}$, $Ly6G^{-}$) in BALF collected from WT, IgHEL, or IgHEL+WT B mice following induction of AAD with Alum + OVA. (B) Flow plots demonstrating B cell phenotype (WT: $IgMa^{-}$, or HEL specific $IgMa^{+}$) in WT, IgHEL, and IgHEL mice that received WT B cell transfer.

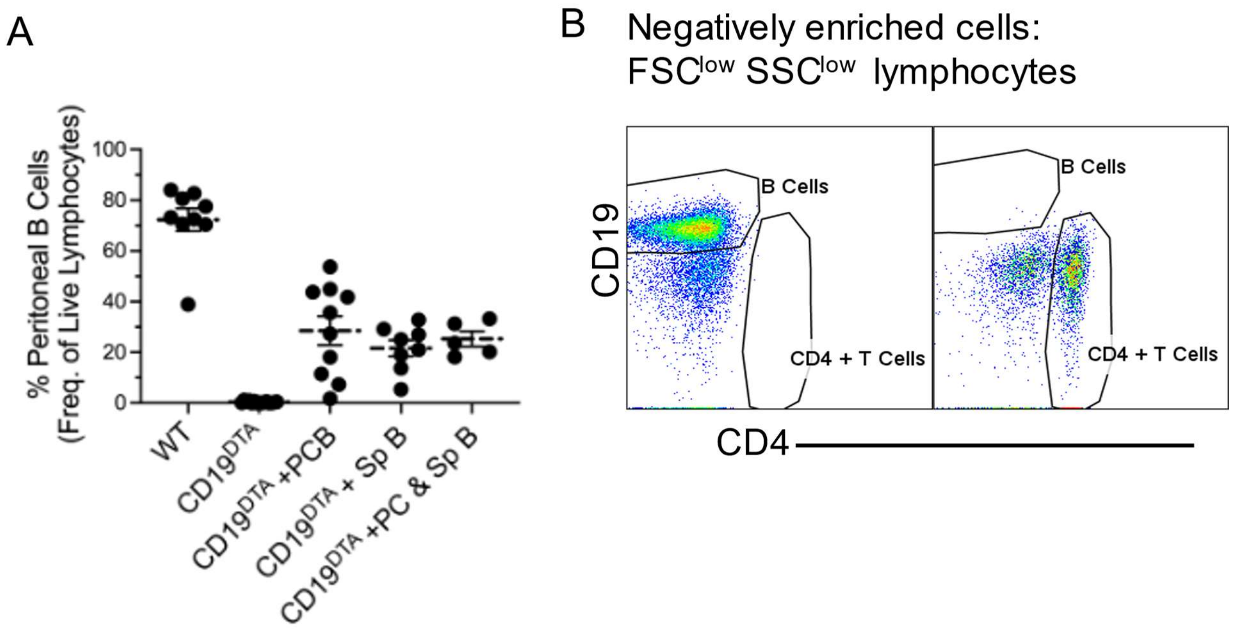

Figure S4. (A) Scatter plot displaying data demonstrating that the adoptive transfer of WT B cells in CD19^{DTA} mice reconstitutes the B cell niche. (B) Flow dot plots demonstrating purity of negatively enriched B and CD4+ T cell fractions used to reconstitute mice.

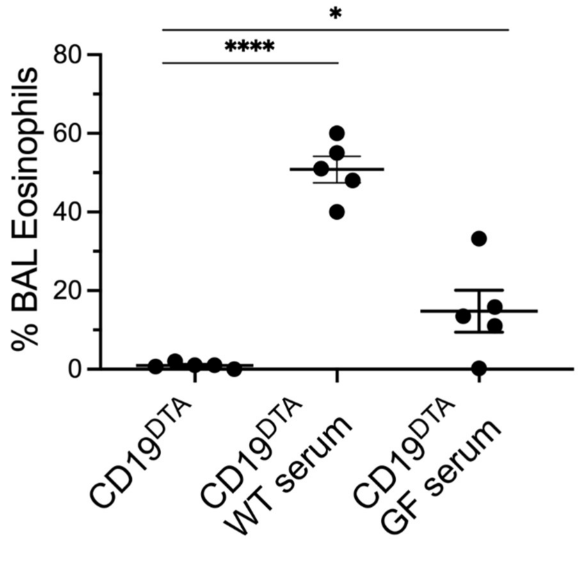

Figure S5. ***Adoptive transfer of naïve WT serum and naïve Germ Free mouse serum rescues the development of Alum-OVA AAD in B cell deficient CD19^{DTA} mice.*** Scatter plot representing the frequency of eosinophils in BALF of mice following induction of Alum-OVA AAD. Error bars indicate mean and standard error of the mean. Each point represents data from an individual animal, with data pooled from 2 independent experiments per graph. Statistical comparisons were performed in GraphPad Prism using a Kruskal Walis ANOVA on Ranks followed by a Dunn's *post hoc* test for multiple comparisons to control. Asterisks denote statistical significance with a p value of less than 0.5, ns indicates no statistical significance.
